# Diagnostic and Prognostic Biomarkers in Renal Clear Cell Carcinoma

**DOI:** 10.3390/biomedicines10112953

**Published:** 2022-11-17

**Authors:** Chaston Weaver, Khaled Bin Satter, Katherine P. Richardson, Lynn K. H. Tran, Paul M. H. Tran, Sharad Purohit

**Affiliations:** 1 Center for Biotechnology and Genomic Medicine, Medical College of Georgia, Augusta University, 1120 15th St., Augusta, GA 30912, USA; 2 Department of Interdisciplinary Health Science, College of Allied Health Sciences, Augusta University, 1120 15th St., Augusta, GA 30912, USA; 3 Department of Urology, Baylor College of Medicine, Houston, TX 76798, USA; 4 Department of Internal Medicine, Yale School of Medicine, Yale University, New Haven, CT 06510, USA; 5 Department of Undergraduate Health Professionals, College of Allied Health Sciences, Augusta University, 1120 15th St., Augusta, GA 30912, USA; 6 Department of Obstetrics and Gynecology, Medical College of Georgia, Augusta University, 1120 15th St., Augusta, GA 30912, USA

**Keywords:** clear cell carcinoma, molecular pathology, biomarkers, gene and protein signatures, machine learning, treatment decision

## Abstract

Renal clear cell carcinoma (ccRCC) comprises over 75% of all renal tumors and arises in the epithelial cells of the proximal convoluted tubule. Molecularly ccRCC is characterized by copy number alterations (CNAs) such as the loss of chromosome 3p and *VHL* inactivation. Additional driver mutations (*SETD2*, *PBRM1*, *BAP1*, and others) promote genomic instability and tumor cell metastasis through the dysregulation of various metabolic and immune-response pathways. Many researchers identified mutation, gene expression, and proteomic signatures for early diagnosis and prognostics for ccRCC. Despite a tremendous influx of data regarding DNA alterations, gene expression, and protein expression, the incorporation of these analyses for diagnosis and prognosis of RCC into the clinical application has not been implemented yet. In this review, we focused on the molecular changes associated with ccRCC development, along with gene expression and protein signatures, to emphasize the utilization of these molecular profiles in clinical practice. These findings, in the context of machine learning and precision medicine, may help to overcome some of the barriers encountered for implementing molecular profiles of tumors into the diagnosis and treatment of ccRCC.

## 1. Introduction

Renal cell carcinoma (RCC) originates in the renal cortex and comprises 80–85% of all primary renal neoplasms [1]. RCC accounts for 2% of global cancer diagnoses and is one of the ten most common types of cancer diagnosed in the United States [2]. In recent years, RCC has become one of the fastest-growing cancers in North America, with the incidence doubling from 1975 to 2016 [2]. According to recent Surveillance, Epidemiology, and End Results Program (SEER) statistics, mortality rates remained relatively stable from 1975 to 2016, which may be associated with improved diagnostic and prognostic measures [2,3]. Despite the tremendous advancements, particularly in targeted therapeutics, RCC remains the most lethal urogenital cancer with a 5-year survival rate of roughly 76% [2,3]. However, the survival statistics depend highly on the initial stage at diagnosis, with localized patients having 93% 5-year survival, while distant cases have only 15.3% [3]. The major subtypes of RCC include clear cell carcinoma (ccRCC, ~75% cases), papillary cell (pRCC, ~10–15% cases), and chromophobe (chRCC, ~5% cases), and other rare types [4]. Each of these types arises from histologically distinct cells [4]. Each subtype arises from a series of complex genetic driver events and molecular aberrations [4]. Over the years, our knowledge has broadened on genetic heterogeneity, including mutational burden and targetable markers by high throughput assays and sequencing technologies [5,6]. Until the recent development of proteomic signature data, all of the research in RCC biomarker identification has focused on genomic alterations and gene expression signatures, which have various limitations preventing their integration into the clinical practice [7].

Current genomic profiling approaches have limitations, such as small numbers of individual mutations, which are both difficult to target therapeutically and fail to capture phenotypic consequences of aberrant gene expression [7,8]. Transcriptomic analyses suffer from a high degree of variability among expression signatures within individual tumors with the absence of validation of the gene signatures in independent population [9]. With the recent integration of protein signature data, a more robust molecular “landscape” for ccRCC may be revealed as the number of protein signature profiles begins to approach the level of genomic and gene expression data currently available [7]. In this literature review, we provide developments over the past 15 years on proteogenomic characterizations of ccRCC and their implication for targeted therapy development by incorporating DNA mutations, gene expression, and proteomic signature data. Additionally, we provide our comments on the role of machine learning and deep learning algorithms that can improve diagnostic and prognostic measures using big data in RCC.

## 2. RCC Subtypes

Major subtypes of RCC include clear-cell (ccRCC), papillary (pRCC), and chromophobe (chRCC), as mentioned earlier [10] (Table 1). The vast majority of RCC cases are of clear-cell morphology (75%), while pRCC (10%), chRCC (5%), and other unclassified and rare subtypes make up the remainder of renal cancer [11]. Clear cell RCC tumors arise from epithelial cells of the proximal convoluted tubule in the nephron and are histologically confirmed by their abundant lipid and glycogen-rich cytoplasmic droplets [12]. Roughly 2–3% of ccRCC are hereditary, originating from *VHL* disease-induced renal neoplastic cysts [13,14]. Hereditary and sporadic tumors alike may degenerate into malignant tumors as the result of a combination of early driver and somatic mutations, DNA methylation, and copy number alterations (CNAs) [14]. These molecular changes promote oncogenesis through the proliferation of a multitude of growth factors and dysregulated pathways, i.e., VEGF, PDGF, and HIF pathways [15]. PRCC tumors are histologically classified as type 1 or type 2, which have distinct molecular and survival differences [16] (Table 1). Most pRCC cases are sporadic; however, type 1 tumors have a hereditary component arising from germline mutations of *MET* [17]. In comparison, type 2 tumors are linked to a greater number of chromosomal aberrations and are associated with higher grade, stage, and an overall worse prognosis [18]. ChRCC tumors are histologically subdivided into a classical type, consisting of pale and eosinophilic cells, and an eosinophilic variant, which contains predominantly eosinophilic cells [19] (Table 1). These tumors are generally viewed as less aggressive compared to the more frequent RCC subtypes [20]. Molecular features unique to chRCC include copy number variations involving complete loss of chromosomes 1, 2, 6, 10, 13, and 17 [19]. Despite these distinctions, much of the current multi-omics analyses have been directed towards ccRCC, as it is the most frequently diagnosed and most lethal subtype [21] (Table 1).

Over 50% of cases of RCC in the clinic are discovered incidentally, showing no common clinical symptoms of flank pain, hematuria, and/or palpable abdominal mass(es), usually associated with RCC [14]. Surgical removal of tumors is the preferred treatment for RCC when patients are in stages I-III; however, up to 1/3 of these patients will experience disease recurrence [22]. For advanced-stage disease, intratumor heterogeneity and tumor clonality are important factors for predicting prognostic outcome [5].

**Table 1:** Renal Cell Carcinoma Types.

RCC Type	Tumor Type	Histology	5-Year Survival (%)	Ref
Clear-Cell	Malignant	Large, lipid-rich cells with abundant cytoplasm	85.5 *	[12,23]
Papillary	Malignant	Type 1: thin, single-cell layered papillae, basophilic, scant cytoplasm; Type 2: thick, large-celled papillae, abundant eosinophilic cytoplasm	90.9	[16,23]
Chromophobe	Malignant	Large cells, distinct membrane, abundant pale cytoplasm	95.2	[19,23]

* 5-year survival for metastatic ccRCC is 12%.

Risk stratification and targeted therapeutic development for these patients have generally relied on certain physiological and biochemical markers expressed within the genome, transcriptome, and proteome [24]. Recent progress in whole genome sequencing techniques has led to the identification of a number of genes with clinical and prognostic relevance for the ccRCC [21]. Additional analysis techniques, such as functional impact mutation ranking, phylogenetic analysis, and ploidy profiling, have revealed distinct driver mutations in the early development of ccRCC tumors [6,25]. The identification of common mutation patterns that initiate tumor progression can improve early detection and prognostication methods, which are two important factors for RCC survival outcomes [11,26].

We queried PubMed and Google Scholar to investigate studies revealing novel gene and protein expression signatures in RCC. PubMed and Google Scholar searches were performed on 21 September 2022, using the keywords “gene expression signature”, “protein expression signature”, and “ccRCC”. The search was restricted to the years 2007–2022. During the literature review, the inclusion criteria consisted of studies reporting sensitivity and specificity (AUROC) values greater than 75% and hazard ratios falling between 0.0–0.5 and >2.0 to capture both protective and detrimental signatures. The exclusion criteria consisted of publications prior to 2007 and studies with discovery sample sizes of less than 40.

## 3. Molecular Changes in RCC

The identification of genomic and transcriptomic biomarkers has added tremendous biological value for ccRCC characterization [27]. The development of ccRCC has been described by a series of molecular changes associated with tumor initiation, driver gene mutations, lethal events, and, ultimately, tumor metastasis [28]. Various DNA alterations are involved in tumor development and progression, including copy number alterations (CNAs), methylation, and mutations that drive genomic instability [28]. The resulting biological state of these alterations is often reflected in the gene expression profiles of tumor cells, from which expression signatures may be identified and associated with clinical metrics, such as diagnosis and prognosis [29]. When analyzed together, the correlation between mRNA transcripts and protein expression for RCC tumors has been shown to be quite variable [27]. As such, protein expression signatures may adequately summarize the consequences of genomic and transcriptomic alterations, while also providing new targetable agents for precision medicine [27].

### 3.1. DNA Alterations

DNA mutations are the most common form of alteration found in all cancers including ccRCC. Genomic alterations in ccRCC are summarized by copy number variations involving whole chromosome alterations (7 and 9), arm-level deletions (3p and 14q) and gains (5q), and additional somatic mutations [30,31]. Many of the mutations associated with ccRCC development follow the two-hit hypothesis of tumorigenesis; the loss of heterozygosity (LOH) occurs via the loss of 3p and inactivation of the remaining allele by somatic mutation [31] (Table 2). The loss of 3p leads to the loss of one copy of *VHL*, *PBRM1*, *SETD2*, and *BAP1*, the most commonly mutated genes in ccRCC. In addition to chromosomal aberrations, promoter CpG hypermethylation, missense, and truncating mutations account for a large percentage of the observed DNA dysregulation [6,31]. The most commonly dysregulated pathways include the well-known VHL/HIF pathway, chromatin remodeling/histone methylation activity, and the PI3K/AKT/mTOR pathway [31] (Table 2). The models for ccRCC development and progression consistently depict the importance of early driver mutations, which leave surroundings cells vulnerable to additional subclonal mutations [26]. Somatic mutations of genes with chromatin remodeling and histone modification capabilities (*PBRM1*, *BAP1*, *SETD2*, *KDM5C*) contribute to increased chromosome instability and alterations in gene expression control, which has been associated with higher-grade tumors and poorer survival [32,33].

Driver mutations are defined as a specific group of mutations that arise in the early stages of cancer and are highly influential in the malignant transformation of tumor cells [34] (Table 2). In certain analyses of the evolution of ccRCC tumor mutations, driver mutations are differentiated by the time at which they occur along phylogenetic trees [5,6]. “Truncal” mutations represent the earliest mutational events in tumor progression, while “branched” mutations occur later and characterize distinct trajectories of the tumor development [5,6]. Despite largely ubiquitous *VHL* inactivation and 3p loss in ccRCC tumors, there is a wide variation in clinical outcomes, which brings into question the role of subclonal and passenger mutations in tumor progression and drug resistance [5,6]. Branched mutations and epigenetic changes often involve gene products associated with chromatin remodeling complexes and hypermethylation, which present unique challenges to the therapeutic targeting [32,35]. Somatic mutations of genes with chromatin remodeling and histone modification capabilities (*PBRM1*, *BAP1*, *SETD2*, *KDM5C*) contribute to increased chromosome instability and alterations in gene expression control, which has been associated with higher grade tumors [32,33] (Table 2). The role of DNA hypermethylation has also been investigated extensively in ccRCC, as silencing of tumor suppressor genes, such as *FBN2*, *PCDH8*, *BNC1*, and *SFRP1*, plays an integral role in the tumor progression [36].

### 3.2. Gene Expression Signatures

The application of gene expression signatures for clinical use has remained a long-standing question since the advent of expression analysis over 20 years ago [37]. Gene expression signatures are defined as a single gene, or group of genes, with an expression pattern that associates with some clinically relevant metric such as diagnosis, prognosis, or predictive treatment response [37]. As a potential biomarker, RNA expression provides a readily and easily available resource for detecting cellular changes reflected in mRNA and other types of extracellular RNA (exRNA) [38]. Extracellular RNA transcripts are also stable in a number of bio-fluids, including urine, serum, and plasma, providing a potentially promising resource for non-invasive collection methods [38]. Changes in gene expression patterns are directly correlated with biologically diseased states and ultimately may represent a surrogate phenotype for the cancer [29]. With recent developments in next-generation sequencing (NGS) transcriptomic signatures can be easily identified. It can also predict splice variants, gene fusions, and epigenetic changes, which are missed in the DNA analysis [39]. Low sample numbers and lack of validation are major obstacles to the clinical transition [40]. The gene expression signatures provided in Table 3 incorporate diagnostic, prognostic, and predictive response outcomes, representing the current state of expression signature analyses.

Classification of RCC, based on gene expression and survival outcomes, was proposed in 2010 as a molecular stratification tool to investigate metastasis and tumor aggressiveness [41,42]. This approach suggested using clear cell type A (ccA) and clear cell type B (ccB) for the classification of RCC to include metastasis and aggressive nature of the RCC tumors [41]. Built off of the ccA/ccB classifiers, ClearCode34 is a prognostic signature that can reliably predict the ccRCC recurrence risk [43]. This gene signature has been shown to identify patients who would benefit from surveillance versus adjuvant therapy following surgery [43]. Gene expression signatures can also be used to differentiate tumor and normal tissue [40]. Therefore, targeting driver mutation-specific expression profiles is a logical strategy to detect early oncogenic changes in pre-neoplastic cells as well as to supplement diagnosis by tumor biopsy and guide treatment decisions [44,45]. Ujfaludi et al. (2022) analyzed the transcriptomic signature of key ccRCC driver genes, *VHL*, *SETD2*, *PBRM1*, and *BAP1*, to find that the median transcription of these genes distinguished ccRCC from normal tissue with a moderate level of sensitivity and specificity (87% and 77%, respectively) [44]. There have been a number of recent clinical trials [46] which have revolutionized the treatment landscape of ccRCC, from which a wealth of biomarker data can be extracted. Biomarker analysis from the recent phase III CheckMate 214 clinical trial compared survival outcomes, progression-free survival (PFS), and overall survival (OS), of combination immune checkpoint inhibitor (ICI) treatment versus sunitinib, with established gene expression signatures [47]. Their findings suggest that combined signatures, such as tumor inflammation with angiogenesis or myeloid changes, may predict better response to immunotherapy versus tyrosine kinase inhibitor (TKI) alone [47]. However, the accumulated wealth of signature data has not been successfully implemented in the clinical setting [48].

One of the greatest barriers to signature implementation in the clinic is reliable data reproducibility, from which further analyses can build upon [49]. In an effort to overcome this, The Cancer Genome Atlas (TCGA) compiled pan-cancer data sets, which have been used as both discovery and validation sets for novel expression signatures [49,50,51]. Other issues in expression signature development include opposing views in the method of analysis, such as “top-down” and “bottom-up” supervised approaches. Supervised, “top-down”, approaches attempt to associate some clinical outcome (survival or metastasis) with an expression profile. Conversely, supervised, “bottom-up”, approaches utilize a biological basis for gene expression, which can be connected to some factors associated with tumor progression [52]. Predictive treatment responses encompass a much smaller range of the available expression signatures, as the individual signatures are tied to specific therapeutic agents [52]. Additionally, expression profiles can represent downstream alterations in proteins which may eventually become therapeutic targets [52].

**Table 3:** List of gene expression studies for diagnostic (ccRCC vs. normal tissue), prognostic (overall survival, recurrence, disease-free survival, and cancer-specific survival), and therapeutic outcomes.

Serial	No. of Genes	Metric	No. of Samples	Measure	Outcome	Ref
1.	3	AUC = 0.912	413	5-year survival post-nephrectomy	Prognostic	[53]
2.	34	RFS: HR = 2.3 (1.6–3.3); CSS: HR = 2.9 (1.6–5.6); OS: HR = 2.4, (1.6–3.7)	530	RFS, CSS, and OS (ccA vs. ccB)	Prognostic	[43]
3.	5	AUC = 0.783	523	Overall Survival (OS)	Prognostic	[54]
4.	16	HR = 3.37	615	RFS, CSS, and OS	Prognostic	[55]
5.	10	HR = 2.85	468	Overall Survival (OS)	Prognostic	[56]
6.	8	AUC = 0.821	888	Fuhrman grade (high grade)	Prognostic	[57]
7.	9	OR = 3.08	443	Recurrence post-nephrectomy, immune signature	Prognostic	[51]
8.	1	AUC = 0.9451	605	Overall Survival (OS) and DFS	Prognostic	[58]
9.	3	AUC = 0.9235–0.9451	605	Normal vs. ccRCC tissue	Diagnostic	[58]
10.	17	HR = 51.37	46	Overall Survival	Prognostic	[50]
11.	4	SN = 87%/SP = 77%	60	Normal vs. ccRCC tissue	Diagnostic	[44]

SN/SP: Sensitivity/Specificity. The names of all genes in the signatures are presented in Supplemental Table S1.

### 3.3. Protein Signatures

Next-generation sequencing (NGS) and other high-throughput analyses provide a wealth of information regarding the abundance of genomic and transcriptomic alterations but often fail to fully characterize the biological state of the tumor as protein modifications are missed at these levels of analysis [59]. Post-translational modifications (PTMs) are enzyme-catalyzed additions of specific functional groups to proteins that promote a variety of biological functions [60]. Certain PTMs, such as phosphorylation and glycosylation, are highly relevant for tumor progression as they regulate cellular processes such as adhesion, migration, signaling, and growth [61,62]. Additionally, PTMs are key events in the dysregulation of metabolic events associated with ccRCC, such as the upregulation of glycolysis and downregulation of the Krebs cycle and electron transport chain, permitting an oncogenic metabolic shift or Warburg effect [11,31]. Proteomic analyses have also been influential in the categorizing of ccRCC, as molecular subgrouping is a useful tool in stratifying patients for precision therapeutics, such as for the use of VEGF inhibitors versus immune-based therapies [30,63]. Despite a multitude of studies over the past 15 years that have uncovered a generalized proteomic profile for ccRCC, none have achieved an adequately comprehensive characterization depicting the necessary linkage between genomic and transcriptomic aberrations, phenotypic presentation, and independent validation to promote clinical utility [59]. Matrix-assisted laser desorption ionization mass spectrometry (MALDI-MS) was the most commonly utilized technique to analyze peptide fractions for diagnostic studies, with validation by western- and immune-blotting (serum/plasma and urine) and immunohistochemistry (IHC) (tissue) (Table 4). The results of these experiments were used in statistical and machine learning models to identify correlation and association with disease vs. control populations. Output metrics are AUC (Area under the receiver operative curve), Sensitivity (SN), Specificity (SP), and hazard ratio (HR). Enrichment analyses were also used to identify upregulation and downregulated pathways in tumor vs. normal tissue. The use of alternate biomarker sources, such as serum/plasma and urine, will likely have important implications for the progression of signatures into clinical practice.

**Table 4:** Studies identifying proteomic signature for clinical management and treatment of renal cell carcinoma patients.

Serial	No. of Proteins	Metric	SN/SP	No. of Samples	Measure	Outcome	Ref
1.	3 ^†^	AUC = 0.90–0.94	-	70	Diagnostic	ccRCC confirmed (NAT vs. tumor)	[64]
2.	3 *	-	88/92%	162	Diagnostic	ccRCC confirmed	[65]
3.	5 *	-	88.80/91.00%	189	Predictive	ccRCC confirmed (recurrence or initial)	[66]
4.	1 *	AUC = 0.86	87/80%	80	Diagnostic	ccRCC confirmed	[67]
5.	1 ^†^	-	96.61/71.43%	881	Prognostic	reduced OS and DFS	[68]
6.	1 **	ACC = 0.930	-	93	Diagnostic	ccRCC vs. Healthy Subjects (HS)	[69]
7.	10 ^†^	AUC = 0.771	-	611	Prognostic	Increased overall survival *	[70]
8.	9 ^†^	AUC = 0.689	-	512	Prognostic	Decreased OS	[71]
9.	7 *	AUC = 0.769	-	445	Prognostic	High/Low-Risk Grouping	[63]
10.	4 ^†,^**	HR = 2.76	-	552	Prognostic	Higher risk of death (decreased OS/RFS)	[72]
11.	20 *	AUC = 0.870	-	232	Prognostic	decreased PFS	[73]

* Serum/Plasma, ^†^ Tissue, ** Urine as an analyte. SN/SP: Sensitivity/Specificity. The list of proteins in the signature(s) are presented in Supplemental Table S2.

Reviews of recent ccRCC proteomic studies revealed a wide variation of dysregulated pathways ranging from metabolic alterations to disruptions of the cell division [64,65]. Clark et al. (2019) characterized the proteogenomic profile of ccRCC by evaluating the role of genomic alterations in promoting the phenotypic presentation of ccRCC tumors. Their efforts, through the Clinical Proteomics Tumor Analysis Consortium (CPTAC), identified a lack of correlation between gene and protein expression, particularly related to oxidative phosphorylation, ribosome, spliceosome, and metabolic pathways [30]. Diagnostic studies found protein signatures associated with protein folding and binding mediation, cell-signaling regulation, tubulin formation, and heat shock protein response in addition to similarities with CPTAC data [64,66,67,68,69] (Table 4). Prognostic protein signatures exhibited dysregulation in the mTORC1 signaling pathway, lipid metabolism, intracellular/vesicle-mediated transport systems, cytokine response and receptor interaction, ribosomal binding proteins (RBPs) functions, as well as a multitude of metabolic and biosynthetic pathways [63,70,71,72,73,74] (Table 4).

Tumor tissue generally contains higher protein concentration than normal tissue, however, the investigation of blood serum/plasma as well as the urine secretome, offer alternative methods for noninvasive biofluid analysis [75]. Tissue-based sampling represents the most direct route of protein extraction, where fresh frozen (FF) tissues, obtained via fine needle aspiration (FNA) biopsy or surgical resection, and formalin-fixed paraffin-embedded (FFPE) tissues are most commonly used for analysis [75,76]. Limited access to biopsy samples is invasive and challenging, while urine and blood collection require less technical expertise and are minimally invasive [77]. Serum samples provide an advantageous route of analysis not only for their less-invasive collection methods but also for potentially early predictive and diagnostic detection of the ccRCC [64,67,68,78]. Urine sampling is the least-invasive peripheral fluid analysis technique, and it contains a less-dynamic complement of proteins compared to the highly abundant proteome of plasma and serum, which make identification of lower molecular weight proteins difficult [59,79]. While the direct relationship of urine analysis to kidney dysfunction may offer promising diagnostic capabilities for the slow-to-moderate onset of tumors, orthogonal models of study incorporating multiple sample types will likely be required to develop consistency in the proteomic characterization of ccRCC [80].

## 4. Utility of Molecular Information in Clinical Management and Treatment of RCC

Diagnosis and staging of RCC are currently performed by anatomical evaluation through imaging techniques (MRI, CT) followed by histopathological confirmation [10]. Diagnosis in the early stage is the most important factor for survival, and at presentation, 25% of the patients already have distant metastasis [81]. The symptoms of the classical triad (hematuria, flank pain, and abdominal mass) are seen in only 10% of the patients’ [2]. Therefore, an early diagnosis of tumor can play a significant role in the survival of the patients. The germline and driver gene mutations can be captured using gene signature assays for diagnostic purposes in both tumor and pre-neoplastic cells [34] (Table 3). Moreover, gene and protein signatures can be used in small renal mass biopsies to identify benign, malignant, and normal tissue to direct therapy [82]. Changes in genetic and epigenetic profiles can also be detected in the patient’s serum and urine analysis for methylation, miRNA, and lncRNA. Genetic markers such as *VHL* [69,71], *APC* [83,84,85], and *P16* [71,83,84,85] are most commonly mentioned in the literature seen in both serum and urine samples in the form of cell-free DNA/ RNA/ methylomes. These tests are non-invasive and can be an excellent screening tool for RCC detection as well as are commonly used in biomarker panels to increase the accuracy of tumor detection. For example, Nuzzo et al. recently presented 300 differentially expressed methylomes in a study with 148 patients for the detection of ccRCC and pRCC with AUROC of 0.99 in serum and 0.86 in urine (patient vs. control). Similarly, miRNA [86] and lncRNA panels [87] are also available for the detection of RCC in urine and serum, respectively. Screening with such tools in high-risk patients can help to identify the tumors early in Stages I-III, where surgical intervention is curative. For imaging, machine learning and deep learning models with the neural network can be used in image classification to identify smaller tumors in suspecting cases [88].

CcRCC treatment depends on the stage at the time of diagnosis. Surgical interventions, such as partial or radical nephrectomy, can be curative in Stages I-III, but about 33% of patients eventually recur. Initial systemic therapy, for locally advanced or metastatic disease, is immune checkpoint inhibitors (ICI) in various combinations of PD-1 (nivolumab & pembrolizumab), PD-L1 (avelumab and atezolizumab), anti-CTLA-4 (ipilimumab) with or without the combination of VEGF inhibitors (axitinib, sunitinib, pazopanib, bevacizumab, etc.), and mTOR inhibitors (everolimus) [89]. The selection of therapy depends on risk stratification using IMDC risk stratification criteria. Response to ICI depends on the expression of many different markers, including the following: PD-L1 [70], tumor-infiltrating lymphocytes [90], tumor mutation burden [91,92], mismatched repair [93], *PTEN* inactivation [94], *POLE* mutation [95], co-mutation of *KRAS* and *STK11* [96], and EGFR mutation [97]. There are other markers in peripheral blood, such as neutrophil to lymphocyte ratio, LDH, peripheral immune cells, circulating tumor DNA, soluble PD-L1, peripheral blood T-cell receptor, and peripheral cytokine [98]. Immune-related factors, such as Beta 2 microglobulin, B7-H4, TOX, and gut microbiota, also play a role in the prediction of ICI response [98].

Prognostic signatures using specific biomarkers (genes and proteins, Table 3 and Table 4) can stratify the patients into groups. These groups can then be used for targeting specific genomic aberrations for predicting drug responses [32]. The best example of this approach is the Prosigna breast cancer signature [99] which quantifies gene expression for 50 genes and uses that information to predict the recurrence [100]. Another example is the use of cytogenetics for diagnosis/stratification of leukemia and lymphomas patients with high and low risk and to identify minimal residual disease [101], i.e., the mutation in *FLT3* gene causes an aggressive form of acute myeloblastic leukemia which is likely to relapse [102]. Similar methods could also be used in ccRCC using mutational, transcriptomic, and proteomic profiling. With metabolic dysfunction being central to tumor progression and aggressiveness in nearly all cancers, including ccRCC, can provide a potential prognostic tool based on metabolic signatures [35]. TCGA analysis found survival outcomes are associated with alternations of mRNA and miRNA expression of multiple metabolic pathways, including glycolysis, Krebs cycle, pentose phosphate pathway (PPP), fatty acid synthesis, PI3K, and AMPK pathway [26]. Dynamic changes to the tumor microenvironment occur in ccRCC development as well as in response to systemic therapies, and clinical trial data have been evaluated to better stratify patients to specific treatments based on gene expression data [9,36,37,38]. Integration of machine learning can identify complex relationships between the gene-gene and gene-protein interactions in regard to survival, recurrence, and treatment responses. The drawback is that overfitting or underfitting the data can lead to false discovery. To overcome that, larger validation studies are needed in a clinical trial controlling all the variables. The eventual application of such signatures in the clinic can guide the physicians for early diagnosis and prognosis to stratify the patients to be treated aggressively or conservatively.

## 5. Conclusions

**Figure 1:**
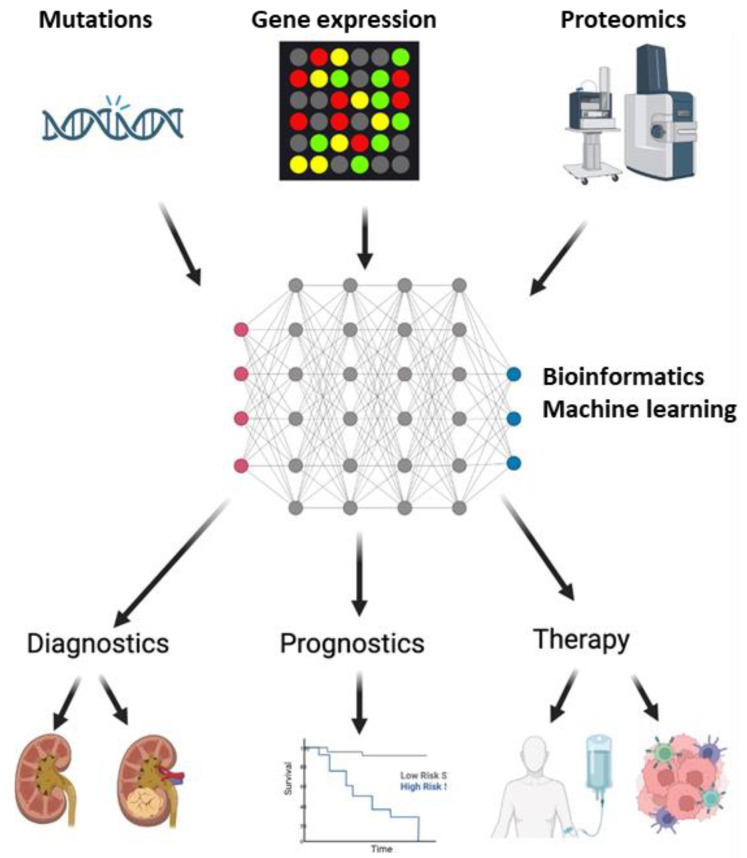
Implementation of biomarkers in Clear Cell Renal Carcinoma (ccRCC). DNA sequencing, microarray, and mass spectrometry with liquid chromatography identify the mutation, gene, and protein expression profiles. These profiles create big data, that, when analyzed by machine learning algorithms, can identify markers for diagnosis, prognosis, and therapeutic decisions for ccRCC. In terms of diagnosis, these biomarkers can help in distinguishing ccRCC patients from healthy individuals, as well as from other patients with benign or malignant renal masses. Disease progression and survival, as an outcome of therapy, can be monitored by prognostic biomarkers. Finally, these biomarkers can provide information, which can offer precision medicine for patients.

A lot of progress has been made in the fields of genomics and proteomics for the high-throughput discovery of novel biomarkers for ccRCC, connecting genomic and molecular data to biological significance. These biomarkers are measured in the tumor tissue itself, serum, or urine and have generated large-scale data. This data can extensively be used for molecular characterization for diagnostic and prognostic signatures. This profiling can identify the tumors in high-risk cases for early diagnosis and predict the prognosis of the patients (Figure 1). The drawback is the lack of validation in a larger multi-institutional cohort in randomized clinical trials. With the validation and implementation of these signatures, it is possible to screen the general population for renal tumors, provide curative therapy in early stages, and predict response and recurrence rates. This would inadvertently lower the physical and emotional burden of the patients as well as the economic burden for society.

## Figures and Tables

**Table 2:** Driver Mutations for ccRCC (Clark et al. [30]).

Gene	Mutation Type	% in ccRCC	Affected Pathway
*VHL*	Missense	45.8	HIFα inhibition
*PBRM1*	Missense, Truncating	32.6	Chromatin remodeling
*SETD2*	Missense, Truncating	11.7	Chromatin remodeling
*BAP1*	Missense, Truncating	9.1	Chromatin remodeling
*KDM5C*	Missense, Truncating	5.5	Chromatin remodeling
mTORC1-associated genes *	Missense, Truncating	13.4	mTORC1 regulation

* MTOR, PTEN, PIK3CA, TSC1.

## Data Availability

Not applicable.

## Supplementary Materials

The following supporting information can be downloaded at: https://www.mdpi.com/article/10.3390/biomedicines10112953/s1, Table S1: Supplementary Table S1: Gene symbols with correlating serial number as presented in Table 3; Table S2: Supplementary Table S2: Protein names and symbols with correlating serial number as presented in Table 4.
