# Supplementary material for: Diagnostic and Prognostic Biomarkers in Renal Clear Cell Carcinoma
Source: Biomedicines. 2022 Nov 17;10(11):2953. doi: 10.3390/biomedicines10112953 (PMC9687861; doi:10.3390/biomedicines10112953)
Supplement: Supplementary file 1 [file biomedicines-10-02953-s001.zip › biomedicines-2027277-supplementary.pdf]

Supplementary information for Weaver et al. Diagnostic and Prognostic Biomarkers in Renal Clear Cell Carcinoma

Table of contents Page

Supplementary Table S1: Gene symbols and correlating serial number as presented in Table 3. ………………………………………………………….. 2

Supplementary Table S2: Protein names and symbols with correlating serial number as presented in Table 4. ……………………………………………………… 3

Supplementary Table S1: Gene symbols with correlating serial number as presented in Table 3.

Serial	Gene Symbol
1.	*VCAM1, EDNRB, RGS5*
2.	*MAPT, STK32B, FZD1, RGS5, GIPC2, PDGFD, EPAS1, MAOB, CDH5, TCEA3, LEPROTL1, BNIP3L, EHBP1, VCAM1, PHYH, PRKAA2, SLC4A4, ESD, TLR3, NRP1, C11orf1, ST13, ARNT, C13orf1, SERPINA3, SCLA3, MOXD1, KCNN4, ROR2, FLJ23867, FOXM1, UNG2, GALNT10, GALNT4*
3.	*CKAP4, ISPD, MAN2A2, OTOF, SLC40A1*
4.	*APOLD1, EDNRB, NOS3, PPAP2B, EIF4EBP1, TUBB2A, LMNB1, CEACAM1, CX3CL1, CCL5, IL6, AAMP, ARF1, ATP5E, GPX1, RPLP1*
5.	*COL1A1, COL5A1, COL11A1, FN1, ICAM1, ITGAL, ITGAM, ITGB2, THBS2, and TIMP1*
6.	*ATOH8, ATP1A3, CNGA1, CHMP4C, PLA2G15, PPP1R1A, SPOCK1, NCRNA00116*
7.	*CD8, CD3, GZMA/B, PRF1, IDO1, CTLA4, PDL1, ICOS, TIGIT*
8.	*AURKB*
9.	*KITLG, AURKB, DLG2*
10.	*IL6, G6PD, TALDO1, POLD4, SQSTM1, CP, DBN1, TMEM8A, TBC1D7, SERPINA3, GIPC1, BAP1, TKT, TLCD1, SLC4A3, PKM, MTX1*
11.	*PBRM1, SETD2, BAP1, VHL*

Supplementary Table S2: Protein names and symbols with correlating serial number as presented in Table 4.

Serial	Protein Name/Symbols
1.	Vimentin, alpha-enolase, H2AFX
2.	RBP6, TUBB, ZFP3
3.	Bcl-2-L, WAP four-disulfide core domain protein*, Krueppel-like factor 8*, MCP-1, ABPP
4.	HSC71
5.	PDZK1
6.	RKIP/p-RKIP
7.	ANK3, CD44, CGN, CHGA, DQX1, IGF2BP2, IGF2BP3, PABPC1L, KIAA1324, RPL22L1
8.	EIF4A1, RPL36A, EXOSC5, RPL28, RPL13, RPS19, RPS2, EEF1A2, OASL
9.	ACC1, AR, MAPK, PDK1, PEA15, SYK, BRAF
10.	PLOD2, FERMT3, SPARC, SIRPα
11.	CD300A, SPP1, DIABLO, HM13, LCP1, SF3B5, TIMM44, LOX, HK2, KRT14, KIF2A, IL18, ANGPTL4, NDRG1, P4HB, GUSB, DNM3, VAV1, HSP90B1, NSA2

*Full names were provided in the original reference, acronyms were not available for these two proteins
